# Inhibitory Effect of Camptothecin against Rice Bacterial Brown Stripe Pathogen *Acidovorax avenae* subsp. *avenae* RS-2

**DOI:** 10.3390/molecules21080978

**Published:** 2016-07-27

**Authors:** Qiaolin Dong, Ju Luo, Wen Qiu, Li Cai, Syed Ishtiaq Anjum, Bin Li, Mingsheng Hou, Guanlin Xie, Guochang Sun

**Affiliations:** 1 College of Plant Science & Technology, Huazhong Agricultural University, Wuhan 430070, China; dongqiaol4321@sina.com (Q.D.); mingshenghou@mail.hzau.edu.cn (M.H.); 2 State Key Laboratory of Rice Biology, China National Rice Research Institute, Hangzhou 310006, China; luojuinsect@sohu.com; 3 State Key Laboratory of Rice Biology, Institute of Biotechnology, Zhejiang University, Hangzhou 310029, China; wenwen20101010@163.com (W.Q.); ishtiaq@kust.edu.pk (S.I.A.); glxie@zju.edu.cn (G.X.); 4 Department of Zoology Kohat University of Science and Technology Kohat, Khyber Pakhtunkhwa 26000, Pakistan; 5 State Key Laboratory Breeding Base for Zhejiang Sustainable Pest and Disease Control, Institute of Plant Protection and Microbiology, Zhejiang Academy of Agricultural Sciences, Hangzhou 310021, China; sungc01@sina.com

**Keywords:** antibacterial activity, antibacterial mechanism, CPT, Hcp, gene expression

## Abstract

Camptothecin (CPT) has anticancer, antiviral, and antifungal properties. However, there is a dearth of information about antibacterial activity of CPT. Therefore, in this study, we investigated the inhibitory effect of CPT on *Acidovorax avenae* subsp. *avenae* strain RS-2, the pathogen of rice bacterial brown stripe, by measuring cell growth, DNA damage, cell membrane integrity, the expression of secretion systems, and topoisomerase-related genes, as well as the secretion of effector protein Hcp. Results indicated that CPT solutions at 0.05, 0.25, and 0.50 mg/mL inhibited the growth of strain RS-2 in vitro, while the inhibitory efficiency increased with an increase in CPT concentration, pH, and incubation time. Furthermore, CPT treatment affected bacterial growth and replication by causing membrane damage, which was evidenced by transmission electron microscopic observation and live/dead cell staining. In addition, quantitative real-time PCR analysis indicated that CPT treatment caused differential expression of eight secretion system-related genes and one topoisomerase-related gene, while the up-regulated expression of *hcp* could be justified by the increased secretion of Hcp based on the ELISA test. Overall, this study indicated that CPT has the potential to control the bacterial brown stripe pathogen of rice.

## 1. Introduction

Camptothecin (CPT), a monoterpenoid indole alkaloid, exhibits promising anticancer and antimicrobial properties [1,2,3]. Several studies have found that CPT has potential inhibitory activities against many kinds of fungi, such as *Rhizoctonia solani*, *Sphaerotheca fuliginea*, *Pseudoperonospora cubensis*, and yeast [1,4]. However, the anticancer and antimicrobial mechanism of CPT was not clearly elucidated until studies showed the inhibitory effect of CPT on the activity of nuclear protein topoisomerase I (Topo I) [5,6]. Topo I is a nuclear enzyme that catalyzes the breakage and rejoining of DNA backbone bonds, which is required for DNA transcription, synthesis, and replication [7]. Furthermore, a cellular poison complex, Topo I-CPT, forms in eukaryotic cells when CPT permeates into them [8].

Little information is available about the antibacterial activity of CPT against plant pathogenic bacteria, in particular *Acidovorax avenae* subsp. *avenae* (Aaa), the pathogen of rice bacterial brown stripe, which has received increased attention in China in recent years [9,10,11,12,13,14,15,16,17,18,19]. Moreover, this pathogenic bacterium has also been reported to be able to infect corn, oats, sugarcane, millet, and foxtail [20]. Due to its ability to spread over long distances through contaminated seeds [9,10,11,12,13,14,15,16,17,18,19,20,21], the need to develop an effective control method for the management of this pathogen is on the rise in rice-growing countries of the world.

Fortunately, genome-wide in silico analysis has identified six topoisomerase-related genes and a large number of secretion system-related genes in Aaa strain RS-2. The topoisomerase-related genes have been found to function in the response of fungal pathogen to CPT [5,6,7,8]. Furthermore, our previous studies have indicated that the secretion system, in particular T6SS, is closely associated with bacterial virulence and its resistance to various stressors, which functions through the secretion of effector protein Hcp or vgrG [12,13,18,22]. Therefore, it is of interest to investigate the involvement of topoisomerase- and secretion system-related genes in the response of Aaa strain RS-2 to CPT.

The aim of this study was to examine the in vitro antibacterial effect of CPT on Aaa strain RS-2 through growth measurement, transmission electron microscopic observation, live/dead cell staining, ELISA tests, and real-time quantitative PCR analysis.

## 2. Results and Discussion

### 2.1. Antibacterial Activity of CPT

**Figure 1:**
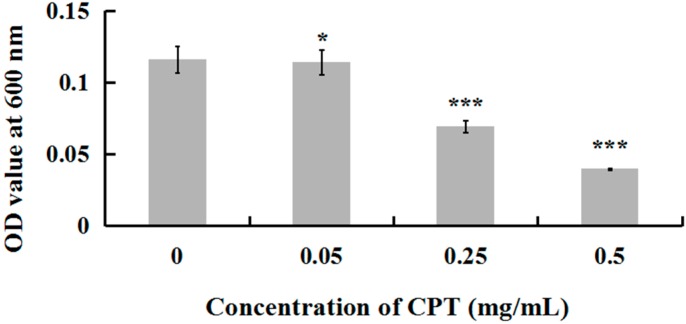
Effect of CPT at different concentrations on the antibacterial activity of *Acidovorax avenae* subsp. *avenae* strain RS-2. *: *p* < 0.05; ***: *p* < 0.001. Error bars represent the standard error of the mean (*n* = 6). Data are from a representative experiment repeated twice with similar results.

To investigate whether CPT has an antibacterial effect, we performed a growth experiment with Aaa strain RS-2. The results indicated that CPT concentrations of 0.05, 0.25, and 0.50 mg/mL caused 18.84%, 40.24%, and 65.72% reduction, respectively, in the OD600 values after 8 h of incubation compared to the control (Figure 1). No inhibitory effect was observed when CPT concentration was lower than 0.01 mg/mL (data not shown). In general, CPT solutions at the three different concentrations showed effective antibacterial activity against Aaa strain RS-2 when compared to the control. This revealed that CPT has potential in controlling rice disease caused by this bacterial pathogen. Nowadays, the application of pesticide in agriculture has been restricted due to the risk that it poses to human health and the environment. In addition, biological control is often affected by changing environmental conditions. The result of this study has provided an alternative to control plant disease. Furthermore, the inhibitory effect of CPT against strain RS-2 increased with an increase in CPT concentration, which was consistent with the result of Mao et al. [23], who found that the antifungal effect was significantly affected by the concentration of CPT.

### 2.2. Effect of Incubation Time on Antibacterial Activity

**Figure 2:**
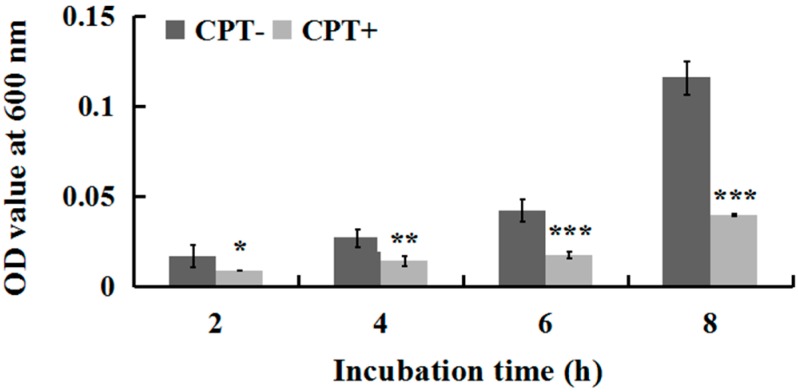
Effect of incubation time on the antibacterial activity of CPT against *Acidovorax avenae* subsp. *avenae* strain RS-2. The concentration of CPT is 0.50 mg/mL. *: *p* < 0.05; **: *p* < 0.01; ***: *p* < 0.001. Error bars represent the standard error of the mean (*n* = 6). Data are from a representative experiment repeated twice with similar results.

To investigate whether incubation time influences the antibacterial activity of CPT, we performed an experiment of growth kinetics with Aaa strain RS-2. The results indicated that the cell number of strain RS-2 increased with an increase in incubation time. However, the addition of CPT caused 35.40%, 47.06%, 58.08%, and 65.72% reduction in the OD600 values of Aaa strain RS-2 after 2, 4, 6, and 8 h of incubation, respectively, compared to the control (Figure 2). The inhibitory effect of CPT on strain RS-2 increased with an increase in incubation time, which revealed that the antibacterial activity of CPT against strain RS-2 was affected by the incubation time. In general, a CPT concentration of 0.5 mg/mL had a strong inhibitory effect against Aaa strain RS-2 regardless of the incubation time.

### 2.3. Effect of the pH Value on Antibacterial Activity

**Figure 3:**
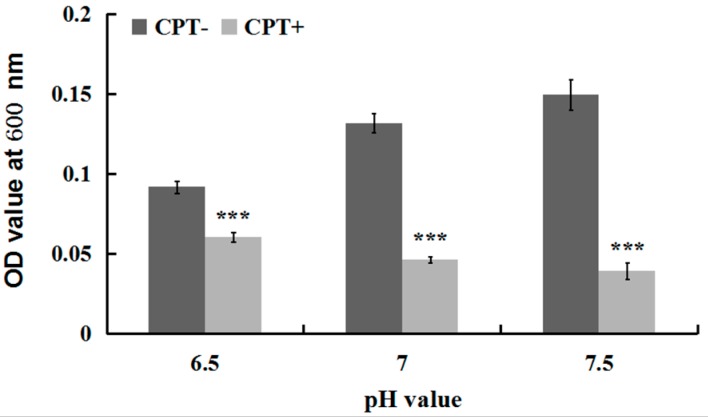
Effect of the pH value on the antibacterial activity of CPT against *Acidovorax avenae* subsp. *avenae* strain RS-2. The concentration of CPT is 0.50 mg/mL, and the incubation time is 8 h. ***: *p* < 0.001. Error bars represent the standard error of the mean (*n* = 6). Data are from a representative experiment repeated twice with similar results.

To investigate whether the antibacterial activity of CPT was affected by acidic or alkaline conditions, we performed an experiment of growth kinetics with Aaa strain RS-2 incubated at different pH values. The results indicated that the addition of CPT caused 34.08%, 64.76%, and 73.89% reduction in the OD600 values of Aaa strain RS-2 at pH values of 6.5, 7.0, and 7.5 respectively, compared to the corresponding controls (Figure 3). Overall, a CPT concentration of 0.5 mg/mL had a significant inhibition on the growth of Aaa strain RS-2 compared to the control regardless of the pH of the half-strength LB broth, suggesting that CPT could be applied in soils with different pH values. Furthermore, the inhibitory effect of CPT at a concentration of 0.5 mg/mL on Aaa strain RS-2 increased with an increase in the pH value of the broth, indicating that CPT had a greater antibacterial activity against strain RS-2 in a mildly alkaline environment than in a mildly acidic environment.

### 2.4. Swarming Motility

**Figure 4:**
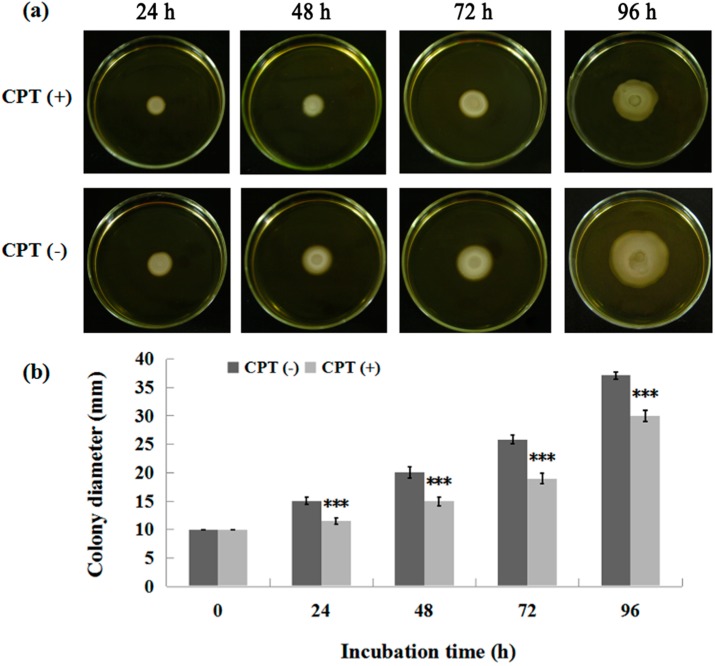
Effect of CPT on the swarming motility of *Acidovorax avenae* subsp. *avenae* strain RS-2. The concentration of CPT is 0.50 mg/mL. ***: *p* < 0.001. Error bars represent the standard error of the mean (*n* = 6). (**a**): Mycelial growth on the PDA; (**b**): Colony diameter at different incubation time.

To investigate whether CPT can affect bacterial movement, we performed an experiment of swarming motility with Aaa strain RS-2. The results indicated that the colony diameter of strain RS-2 is 10.0, 14.5, 20.0, 25.0, and 36.5 mm in the absence of CPT, while the colony diameter of strain RS-2 is 10.0, 11.0, 14.2, 18.0, and 29 mm in the presence of CPT, after 0, 24, 48, 72, and 96 h of incubation time respectively (Figure 4). It is obvious that a CPT concentration of 0.5 mg/mL had a significant inhibition on the swarming motility of Aaa strain RS-2 regardless of the incubation time. Although the colony diameter of strain RS-2 increased with an increase in incubation time, the addition of CPT caused a 24.1%, 29.0%, 28.0%, and 20.5% reduction in the colony diameter compared to the corresponding controls at 24, 48, 72, and 96 h of incubation time, respectively. Interestingly, the swarming motility has been reported to be frequently linked with bacterial growth, propagation, and virulence [14,24]. Therefore, the reduction in swarming motility is considered to be consistent with the antibacterial activity of CPT against Aaa strain RS-2.

### 2.5. Live/Dead Cell Staining

**Figure 5:**
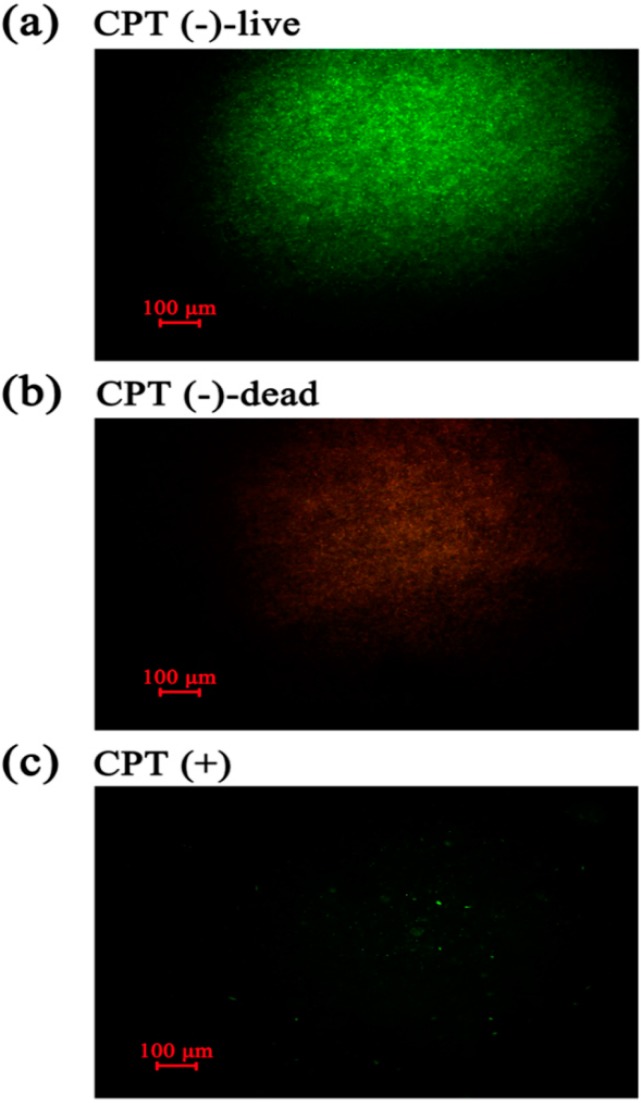
Live/dead cell staining analysis of strain RS-2 cells exposed to 0.5 mg/mL CPT for 8 h. Staining were carried out using live/dead BacLight bacterial viability kit (Invitrogen, Carlsbad, CA, USA), and visualized by fluorescence microscopy. Green fluorescence is representative of live bacteria with intact membranes, while red fluorescence is representative of dead bacteria. (**a**): Live bacteria in negative control; (**b**) Dead bacteria in negative control; (**c**) Bacteria in CPT treatment.

To understand the mechanism underlying the antibacterial activity of CPT, we performed an experiment of live/dead cell staining with cells of Aaa strain RS-2 exposed to CPT for 8 h. The results indicated that in the absence of CPT, live bacteria with intact membranes fluoresce green (Figure 5a), while dead bacteria fluoresce red (Figure 5b). Interestingly, bacterial cells continued to fluoresce green when exposed to CPT (Figure 5c). However, the number of cells was dramatically reduced when bacteria were exposed to CPT, indicating an inhibition of bacterial growth and replication. Therefore, it seems that CPT has a bacteriostatic effect rather than bactericidal effect on strain RS-2.

### 2.6. Transmission Electron Microscopy (TEM)

**Figure 6:**
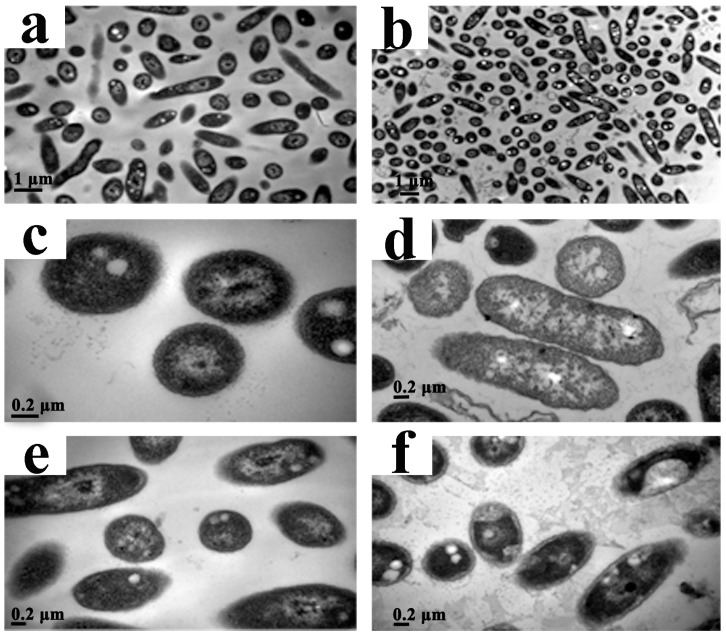
Transmission electron microscopic observation of *Acidovorax avenae* subsp. *avenae* strain RS-2 treated without (**a**,**c**,**e**) and with 0.5 mg/mL of CPT (**b**,**d**,**f**). Scale bar in (**a**,**b**) = 1.0 μm; in (**c**–**f**) = 0.2 μm.

To investigate the effect of CPT on the structural integrity of cells, we performed a TEM analysis with Aaa strain RS-2. The TEM images indicated that the control cells had intact and apparent cell membranes (Figure 6a,c,e). However, after exposure of Aaa strain RS-2 to 0.5 mg/mL of CPT for 8 h, the intracellular substances degraded and some bacterial cells formed vacuole-like structures (Figure 6b,d). Furthermore, the cell surface became rough and abnormal, and there was evidence of cytoplasmic condensation (Figure 6f). In addition, in the presence of CPT there was about a 5.0% increase in release of the intracellular nucleic acids and proteins in Aaa strain RS-2 based on membrane integrity monitoring method [12], justifying the result of the TEM micrographs. Therefore, it could be inferred that CPT caused a slight damage to the bacterial membranes.

Our previous report indicated that the antimicrobial ability of CPT may be at least partially attributed to the lysis of the cytoplasmic membrane [23], although the impedance in the DNA transcription, synthesis, and replication has been regarded as the main antimicrobial mechanism of CPT [25]. Furthermore, membrane damage has been found to be an important event in the inactivation of bacteria by high pressure, but the nature of membrane damage and its relation to cell death may differ between species and phases of growth [26]. In this study, membrane damage affected bacterial growth and replication but did not cause bacterial death, which was confirmed by the results of live/dead cell staining. Therefore, it could be concluded that CPT demonstrated a bacteriostatic action against Aaa strain RS-2.

### 2.7. Secretion of Effector Protein Hcp

**Figure 7:**
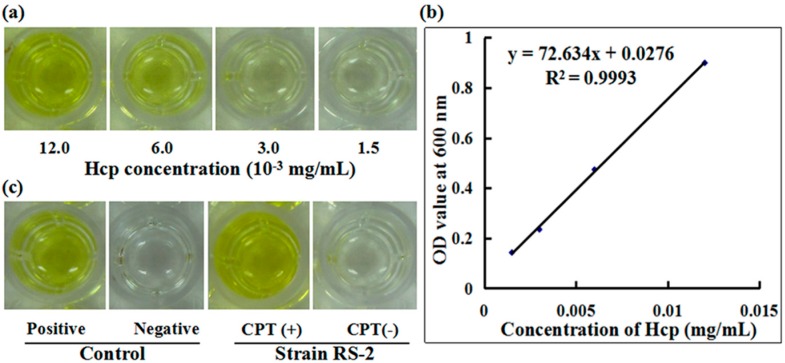
Effect of CPT on the secretion of Hcp protein. (**a**) ELISA of Hcp protein at different concentrations; (**b**) Standard curve; (**c**) ELISA measurement of Hcp secreted by CPT.

To understand the role of T6SS in the response of Aaa strain RS-2 to CPT, we performed an ELISA experiment with the polyclonal antibody of effector protein Hcp. A standard curve of Hcp protein was drawn based on the OD value-concentration of Hcp protein, which was determined using an indirect ELISA method (Figure 7a). In this study, the standard curve of Hcp protein was represented as y = 72.634x + 0.0276 (R^2^ = 0.9993) (Figure 7b). The reliability of the standard curve was justified by the high correlation coefficient in this study. There was a positive ELISA reaction for Hcp protein in the culture broth of strain RS-1 in the presence of 0.5 mg/mL CPT, while a negative ELISA reaction for Hcp protein was observed in culture broth of strain RS-2 in the absence of CPT (Figure 7c). Quantitative analysis indicated that the concentration of Hcp in the CPT treated sample and the positive control is 0.017 and 0.012 mg/mL, respectively, based on the standard curve. The result of this study suggests that the addition of CPT caused an increased secretion of Hcp protein in live bacteria, which resulted from the bacteriostatic effect of CPT, based on the live/dead cell staining analysis of Aaa strain RS-2. Ho et al. [27] found that membrane damage was able to induce the T6SS, therefore, it could be suggested that the increased secretion of Hcp protein may be partially attributed to membrane damage.

### 2.8. Differential Gene Expression

**Figure 8:**
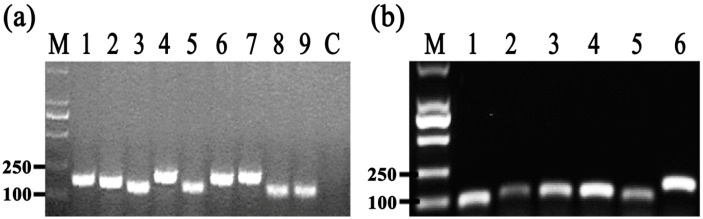
Results of conventional PCR for primers used in qRT-PCR assay. (**a**) M = Marker DL2000 (TaKaRa, Dalian, China); 1 = Lip; 2 = clpA; 3 = TomB; 4 = porin; 5 = protein F; 6 = ImpJ; 7 = ImpG; 8 = hcp; 9 = vgr-1; Control = distilled water (negative control); (**b**) M = Marker DL2000 (TaKaRa, Dalian, China); 1 = topB; 2 = gyrB; 3 = parE; 4 = parC; 5 = topA; 6 = gyrA.

**Figure 9:**
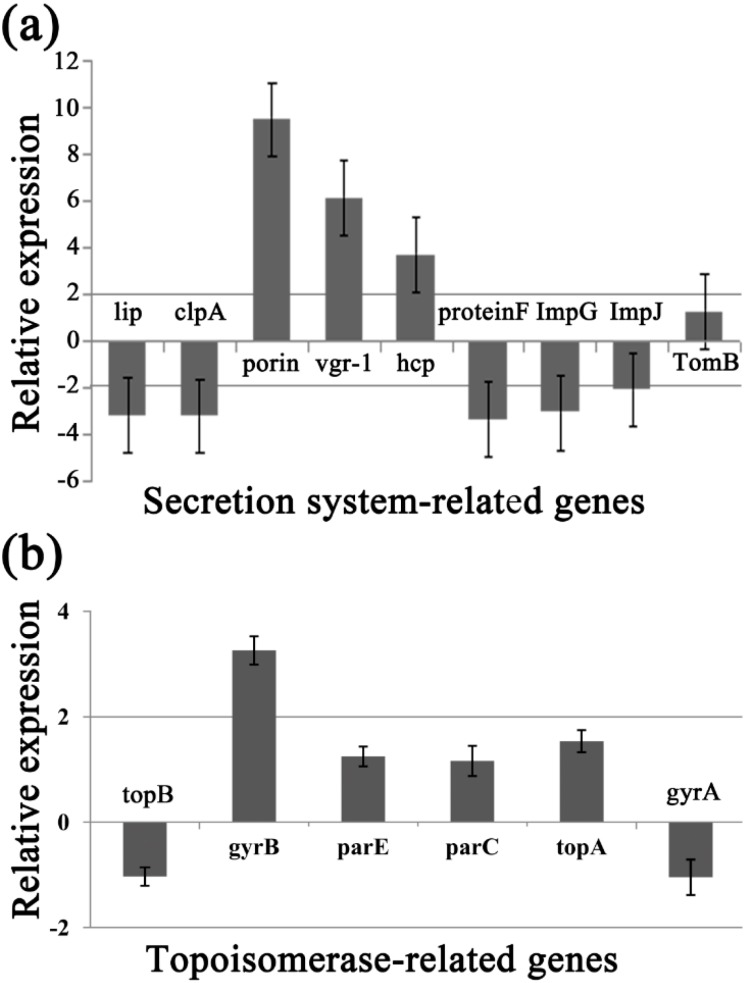
Effect of CPT at 0.5 mg/mL on the expression of secretion system- or topoisomerase-related genes of *Acidovorax avenae* subsp. *avenae* strain RS-2. (**a**) Secretion system-related genes; (**b**) Topoisomerase-related genes. Data are the average value of three replicates with standard error.

To identify the differentially expressed genes involved in the response of Aaa strain RS-2 to CPT, we performed quantitative real-time PCR (qPCR) experiments. The qPCR primers for secretion system- or topoisomerase-related genes were validated in this study using the conventional PCR reaction (Table 1, Figure 8), while the expressions of these genes are illustrated in Figure 9, which revealed that compared to the pathogen control, the addition of CPT caused 3.17, 3.20, 3.36, 3.04, 2.06, 1.03, and 1.04-fold down-regulation in the expression of genes encoding Lip, ClpA, protein F, ImpJ, ImpG, topB, and gyrA; and 9.50, 6.16, 3.72, 1.28, 3.26, 1.25, 1.16, and 1.53- fold up-regulation in the expression of genes encoding porin, vgr-1, hcp, TomB, gyrB, parE, parC, and topA, respectively. The results revealed that CPT caused differential regulation of the listed genes in Aaa strain RS-2.

Among the 6 topoisomerase-related genes, CPT treatment caused a significant differential expression of gyrB gene, which encodes DNA gyrase B-subunit protein, and functions in bacterial transcription and replication processes. Furthermore, the greatest change in expression of secretion system-related genes by CPT was for the porin gene, which has been reported to be associated with bacterial survival and its response to bactericidal compounds [28]. The significant change induced by CPT was also observed in the T6SS genes such as those encoding RND efflux system outer membrane lipoprotein (vgr-1), ATP-dependent Clp protease (ClpA), ImpJ, ImpG, and Lip. Interestingly, T6SS genes have been reported to play an important role in various aspects such as bacterial virulence, survival, biofilm formation, EPS production, and resistance to different environmental stressors [29,30,31,32,33,34,35,36]. In agreement with the down-regulated expression of ClpA in this study, Yang et al. [12] found that the expression of Clp was down-regulated by chitosan. Therefore, it could be inferred that these differentially expressed genes may be involved in the response of Aaa strain RS-2 to CPT.

## 3. Experimental Section

### 3.1. Materials

CPT natural product was purchased from Hubei Widely Chemical Technology Co., Ltd. (Wuhan, China) and was kept in a refrigerator at −20 °C before use. Stock solution of CPT (10 mg/mL) was prepared in 1.0% dimethyl sulfoxide (DMSO) with pH of 7.0, and filtered by 0.22 μm membranes (Millipore, Bedford, TX, USA). Controls were prepared in the same way but without added CPT. Strain RS-2 of *A. avenae* subsp. *avenae* was isolated from natural diseased rice plants in the fields. The bacterial strain was routinely grown and maintained at 30 °C using Luria-Bertani (LB) agar plates as described in strain RS-1 [9].

### 3.2. In Vitro Antibacterial Activity of CPT

CPT solutions of 5 mL were prepared by adding CPT stock to half-strength LB broth to give a final CPT concentration of 0.05, 0.25, and 0.50 mg/mL, respectively, while 1.0% DMSO solution with the same pH was used as the control. The effect of concentration on the antibacterial activity of CPT against Aaa strain RS-2 was evaluated by inoculating 100 μL of an overnight bacterial culture into CPT solutions of different concentrations, and incubating at 30 °C with 160 rpm for 8 h. The effect of incubation time on the antibacterial activity of CPT against Aaa strain RS-2 was examined by inoculating 100 μL of an overnight bacterial culture into CPT solution of 0.50 mg/mL and the mixture was then incubated at 160 rpm for 0, 2, 4, 6, and 8 h, respectively. The effect of pH on the antibacterial activity of CPT against Aaa strain RS-2 was evaluated by inoculating 100 μL of an overnight bacterial culture into CPT solutions of 0.50 mg/mL with a final pH value of 6.5, 7.0, and 7.5, respectively, and incubating at 160 rpm for 8 h. The bacterial numbers were determined according to the method of Cui et al. [13] by measuring the optical density at 600 nm using a Thermo Multiskan EX Micro plate Photometer (Thermo Fisher Scientific Inc., Waltham, MA, USA). Six replicates were prepared for each treatment, and the experiment was repeated twice.

### 3.3. Swarming Motility

Swarming motility of Aaa strain RS-2 was examined on LB broth supplement with 0.7% (*w*/*v*) agar (Difco, Franklin Lakes, NJ, USA) [37]. Bacteria were grown in LB broth overnight at 30 °C. Following centrifugation at 8000 *g* for 2 min at 4 °C, the pellets were washed twice with ddH_2_O, and then resuspended in ddH_2_O up to a final concentration of OD600 of 1.0. Five microliters of bacterial suspension were spotted onto the center of the LB plates (diameter, 90 mm) with and without 0.5 mg/mL CPT. The LB plates were incubated at 30 °C for 4 days. The motility of Aaa strain RS-2 was determined by measuring the migration zones as described previously [14].

### 3.4. Live/Dead Cell Staining

Live/dead (BacLight bacterial viability kit; Invitrogen) staining was carried out on bacterial cells exposed to 0.50 mg/mL CPT. In addition, the validity of this kit was justified by staining of live and dead bacteria, respectively. Live bacteria were directly collected from the negative control, while dead bacteria were obtained by treating the same number of live bacteria with isopropanol according to the instruction of the kit. This kit contains two nucleic acid stains, propidium iodide stain (a red-fluorescent), and SYTO 9 stain (a green-fluorescent). The staining procedure was performed according to the manufactures’ instructions (BacLight bacterial viability kit; Invitrogen). Fluorescence was then observed as described by Cui et al. [38] with an inverted confocal fluorescence microscope (Olympus DP50 BX 51, Tokyo, Japan).

### 3.5. Transmission Electron Microscopy (TEM)

Aaa strain RS-2 was prepared for TEM analysis as described previously [39]. Described briefly, bacteria were inoculated into CPT solution of 0.5 mg/mL to give a final bacterial concentration of 10^8^ CFU/mL and then the mixture was incubated on a rotary shaker (160 rpm) at 30 °C for 8 h. After centrifugation at 8000 *g* for 10 min at 4 °C, bacterial cells were washed twice with 0.1 mol/L phosphate buffered saline (PBS) solution at pH 7.2 and fixed with 2.5% (*v*/*v*) glutaraldehydein. Post-fixation was carried out in 1% (*w*/*v*) osmium tetroxide in 0.1 mol/L PBS for 1 h at room temperature followed by dehydration at 4 °C for 15 min in a graded series of ethanol solutions (70%, 80%, 90%, 95%, and 100%, *v*/*v*) and embedded in Epon812, a low-viscosity embedding medium. Thin sections of the specimens were cut with a diamond knife on an Ultra microtome (Super Nova; Reichert-Jung Optische Werke, Vienna, Austria) and the sections were double-stained with saturated uranyl acetate and lead citrate. The grids were examined with a JEM-1230 transmission electron microscope (Hitachi, Tokyo, Japan) at an operating voltage of 75 kV.

### 3.6. Measurement of the Secreted Hcp by ELISA

Enzyme-linked immune sorbent assay (ELISA) was used for the measurement of the secreted Hcp, which has been found to be involved in the bacterial virulence and resistance to various stressors [13,15,18]. The ELISA was performed in a 96 microtiter plate by pipetting 100 μL of the polyclonal antibody into the wells of the plate [40]. The polyclonal antisera was generated by immunizing rabbits with the Hcp-His fusion protein as described [41]. Following the examination of the sensitivity and specificity [22], the polyclonal antisera at a 1:5000 dilution was used for the detection of Hcp secreted from Aaa strain RS-2 in the presence or absence of 0.5 mg/mL CPT. The absorbance (optical density) of each well was recorded using an ELISA plate reader at 450 nm. The quantity of the secreted Hcp protein was determined based on a standard curve, which was generated by relating the optical density with the concentration of the purified Hcp protein.

### 3.7. RNA Isolation and Real-Time Quantitative PCR

Table 1 presents qRT-PCR primers of 6 topoisomerase-related genes and 9 secretion system-related genes. The selection of these virulence-related gene transcripts was mainly based on the findings of previous studies, which were conducted on strain RS-1 of Aaa [12,13,14,15]. Furthermore, 16S ribosomal DNA (rDNA), an internal control gene, was used as the reference. The accuracy and specificity of the designed qPCR primers were validated by the conventional PCR, which was performed with the following conditions: 95 °C for 30 s; followed by 40 cycles at 95 °C for 5 s and 60 °C for 34 s.

After incubation of Aaa strain RS-2 in 0.5 mg/mL CPT solution for 8 h, bacterial cells were harvested and used for total RNA extraction, which were performed using RNeasy Mini Kit according to the manufacturer’s instruction (QIAGEN Co., Ltd., Shanghai, China). Purified RNAs were suspended in 50–100 μL of 0.1% DEPC (dimethyl-pyrocarbonate)-treated water. RNA concentrations were calculated by measuring absorbance at 260 nm using a Nanodrop 2000C spectrophotometer (Thermo Fisher Scientific, Lanham, MD, USA). The cDNA was synthesized with a TransScript One-Step gDNA Removal and cDNA Synthesis SuperMix (TaKaRa, Dalian, China), which was then used directly as the template for qRT-PCR using a SYBR^®^ Premix Ex Taq™ (Tli RNase HPlus) (TaKaRa, Dalian, China) following the instruction of the kit’s manual on an ABI Prism 7500 sequence detection system (Applied Biosystems, Foster City, CA, USA). Normalized expression levels of the target gene transcripts were calculated relative to the 16S rDNA gene using the ∆∆Ct method, where Ct is the threshold cycle. Each result represents the average of three independent determinations. qRT-PCR was performed with the following conditions: 95 °C for 30 s; followed by 40 cycles at 95 °C for 5 s and 60 °C for 34 s, followed by a standard melting curve stage. Finally, the expression levels of the target gene transcripts were calculated using the 2^−∆∆Ct^ method.

### 3.8. Statistical Analyses

The software STATGRAPHICS Plus, version 4.0 (Copyright Manugistics Group Inc., Rockville, MD, USA) was used to perform the statistical analyses. Levels of significance (*p* < 0.05) of main treatments and their interactions were calculated by an analysis of variance after testing for normality and variance homogeneity.

## 4. Conclusions

Results from this study indicated that CPT solutions at 0.05, 0.25, and 0.50 mg/mL inhibited the growth of Aaa strain RS-2, and the inhibitory effect depends on the concentration, pH, and incubation time. Furthermore, the antibacterial activity of CPT was supported by the result of TEM observations and live/dead cell staining analysis. In addition, qRT-PCR results indicated that exposure of Aaa strain RS-2 to CPT caused differential expression of one topoisomerase-related gene and eight secretion system-related genes, while an increased secretion of Hcp protein in live bacteria was detected by ELISA analysis using Hcp antiserum. Overall, the results of this study revealed that CPT has a potential to control the bacterial brown stripe pathogen of rice.

## Figures and Tables

**Table 1:** Primers of target genes used for quantitative real-time PCR in this study.

Target Genes	Primer Sequence (5′-3′)	Putative Protein Function (s)	Amplication Size (bp)
16S rDNA	F-TTGCGGTCCCCTGCTTTCAT		120
	R-CGGTAACAGGTCTTCGGATGCT		
Secretion system			
clpA	F-CACGATCCAGATCCTCTGCC	ATP-dependent Clp protease	163
	R-GCCCATGTCCAGCGAATAGA		
porin	F-GCACCACCTGGTCCACAACA	Porin Gram-negative type	192
	R-GCGTCGAACATTCCAAGGTG		
protein F	F-CCGCGGGAATTCTCTTCCAT	General secretion pathway protein F T2SF; Type II secretion system (T2SS) reductase	131
	R-CCGATGAGCTTGGCCTTGA		
tomB	F-ATCAACCGCTACCGC	TomB protein interacts with outer membrane receptor proteins that carry out high-affinity binding and energy-dependent uptake of specific substrates into the periplasmic space	129
	R-ATCAAGGTCTGGACCTCGTTGAAGG		
impG	F-GCCACAAGTTCCTTTTGCA	Type VI secretion protein ImpG	181
	R-AAGAACGGCACGAAATCC		
impJ	F-TCCAGGATGCCAACGACA	Type VI secretion protein ImpJ	181
	R-GACCACGGTGGGAATGAA		
lip	F-GCAGTGCGGATGTCCGTACCTT	Type VI secretion lipoprotein	174
	R-TCCTTGCCCACCGTGATGCT		
hcp	F-GACTCCGTGCTGGTGAAT	Type VI secretion system effector	100
	R-TTCCCTTGATGTCGTAGCC		
vgr-1	F-ATCCGATGGAAAAGAAACTC	RND efflux system outer membrane lipoprotein [*Acidovorax citrulli* AAC00-1]	113
	R-AATAGATGCCCTCGTGCT		
Topoisomerase			
topB	F-CTGGACAAGTTCGTGAGCAT	DNA topoisomerase III, *Burkholderia* type	89
	R-AACTCGAAGTTCACCTTGCC		
gyrB	F-CTGCTTCACCAACAACATCC	DNA gyrase subunit B	99
	R-TTCTGCTCGATGTACTTGCC		
parE	F-ATGAACGAACTGGTCCACCT	Topoisomerase IV subunit B	105
	R-GTATTGCCAGGTCTGGGTCT		
parC	F-CAAGATCGAGCAGGAACTCA	Topoisomerase IV subunit A	106
	R-CTCGATCTCCTTGACCATGA		
topA	F-GGGCAGGAACTCTTCCAGTA	DNA topoisomerase IB (poxvirus type)	83
	R-TCGTGCAGGTAGTCGTTCAC		
gyrA	F-GCACGACTACATCCTGTGCT	DNA gyrase subunit A	148
	R-CAGCACCACGTTGATCTTCT		

## References

[1] Li S., Zhang Z., Cain A., Wang B., Long M., Taylor J. (2005). Antifungal activity of camptothecin, trifolin, and hyperoside isolated from *Camptotheca acuminata*. J. Agric. Food Chem..

[2] Ulukan H., Swaan P.W. (2002). Camptothecins: A review of their chemotherapeutic potential. Drugs.

[3] Li Y.Y., Chen S.W., Yang L.M., Wang R.R., Pang W., Zheng Y.T. (2010). The anti-HIV actions of 7- and 10-substituted camptothecins. Molecules.

[4] Zhang L., Sun Y., Wang P., Tong S.M., Ma L.J. (2008). Antifungal activity of camptothecin on *Rhizoctonia solani*, *Sphaerotheca fuliginea* and *Pseudoperonospora cubensis*. J. Zhejiang For. Coll..

[5] Lee J.H., Lee J.M., Kim J.K., Ahn S.K., Lee S.J., Kim M.Y., Jew S.S., Park J.G., Hong C.I. (1998). Antitumor activity of 7-[2-(*N*-isopropylamino) ethyl]-(20*S*)-camptothecin, CKD602, as a potent DNA topoisomerase I inhibitor. Arch. Pharm. Res..

[6] Hsiang Y.H., Liu L.F. (1988). Identification of mammalian DNA topoisomerase I as an intracellular target of the anticancer drug camptothecin. Cancer Res..

[7] Sinha B.K. (1995). Topoisomerase inhibitors. Drugs.

[8] Pommier Y., Leo E., Zhang H., Marchand C. (2010). DNA topoisomerases and their poisoning by anticancer and antibacterial drugs. Chem. Biol..

[9] Xie G.L., Zhang G.Q., Liu H., Lou M.M., Tian W.X., Li B., Zhou X.P., Zhu B., Jin G.L. (2011). Genome sequence of the rice-pathogenic bacterium *Acidovorax avenae* subsp. *avenae* RS-1. J. Bacteriol..

[10] Kakar K., Nawaz Z., Cui Z., Almoneafy A., Zhu B., Xie G.L. (2014). Characterizing the mode of action of *Brevibacillus laterosporus* B4 for control of bacterial brown strip of rice caused by *A. avenae* subsp. *avenae* RS-1. World J. Microbiol. Biotechnol..

[11] Li B., Liu B.P., Yu R.R., Tao Z.Y., Wang Y.L., Xie G.L., Li H., Sun G.C. (2011). Bacterial brown stripe of rice in soil-less culture system caused by *Acidovorax avenae* subsp. *avenae* in China. J. Gen. Plant Pathol..

[12] Yang C.L., Li B., Ge M.Y., Zhou K.L., Wang Y.L., Luo J., Ibrahim M., Xie G.L., Sun G.C. (2014). Inhibitory effect and mode of action of chitosan solution against rice bacterial brown stripe pathogen *Acidovorax avenae* subsp. avenae RS-1. Carbohydr. Res..

[13] Cui Z.Q., Jin G., Li B., Kakar K.U., Ojaghian M.R., Wang Y.L., Xie G.L., Sun G.C. (2015). Gene expression of type VI secretion system associated with environmental survival in *Acidovorax avenae* subsp. *avenae* by principle component analysis. Int. J. Mol. Sci..

[14] Liu H., Tian W.X., Ibrahim M., Li B., Zhang G.Q., Zhu B., Xie G.L. (2012). Characterization of *PilP*, a gene required for twitching motility, pathogenicity, and biofilm formation of *Acidovorax avenae* subsp. *avenae* RS-1. Eur. J. Plant Pathol..

[15] Ibrahim M., Shi Y., Qiu H., Li B., Jabeen A., Li L., Liu H., Kube M., Xie G.L., Wang Y.L. (2012). Differential expression of in vivo and in vitro protein profile of outer membrane of *Acidovorax avenae* subsp. *avenae*. PLoS ONE.

[16] Li B., Wang L., Ibrahim M., Ge M.Y., Wang Y.L., Mannan S., Asif M., Sun G.C. (2015). Membrane protein profiling of *Acidovorax avenae* subsp. *avenae* under various growth conditions. Arch. Microbiol..

[17] Liu H., Yang C.L., Ge M.Y., Ibrahim M., Li B., Zhao W.J., Chen G.Y., Zhu B., Xie G.L. (2014). Regulatory role of *tetR* gene in a novel gene cluster of *Acidovorax avenae* subsp. *avenae* RS-1 under oxidative stress. Front. Microbiol..

[18] Li B., Ibrahim M., Ge M.Y., Cui Z.Q., Sun G.C., Xu F., Kube M. (2014). Transcriptome analysis of *Acidovorax avenae* subsp. *avenae* cultivated in vivo and co-culture with *Burkholderia seminalis*. Sci. Rep..

[19] Ge M.Y., Li B., Wang L., Tao Z.Y., Mao S.F., Wang Y.L., Xie G.L., Sun G.C. (2014). Differentiation in MALDI-TOF MS and FTIR spectra between two pathovars of *Xanthomonas oryzae*. Spectrochim. Acta A Mol. Biomol. Spectrosc..

[20] Song W., Kim H., Hwang C., Schaad N. (2004). Detection of *Acidovorax avenae* ssp. *avenae* in Rice Seeds Using BIO-PCR. J. Phytopathol..

[21] Shakya D., Chung H. (1983). Detection of *Pseudomonas avenae* in rice seed. Seed Sci. Technol..

[22] Li B., Ge M.Y., Zhang Y., Wang L., Ibrahim M., Wang Y.L., Sun G.C., Chen G.Y. (2016). New insights into virulence mechanisms of rice pathogen *Acidovorax avenae* subsp. *avenae* strain RS-1 following exposure to *ß*-lactam antibiotics. Sci. Rep..

[23] Mao S.F., Luo J.Y., Wu Z.Y., Lei P., Ge M.Y., Li B., Wang Y.L., Zhang L.Q., Sun G.C. (2015). Physiological and morphological changes in *Botrytis cinerea* induced by the plant alkaloid camptothecin. Trop. Plant. Pathol..

[24] Connelly M.B., Young G.M., Sloma A. (2004). Extracellular proteolytic activity plays a central role in swarming motility in *Bacillus subtilis*. J. Bacteriol..

[25] Lorence A., Nessler C.L. (2004). Camptothecin, over four decades of surprising findings. Phytochemistry.

[26] Pagán R., Mackey B. (2000). Relationship between membrane damage and cell death in pressure-treated *Escherichia coli* cells: Differences between exponential- and stationary-phase cells and variation among strains. Appl. Environ. Microbiol..

[27] Ho B.T., Basler M., Mekalanos J.J. (2013). Type 6 secretion system-mediated immunity to type 4 secretion system-mediated horizontal gene transfer. Science.

[28] Achouak W., Heulin T., Pages J.M. (2001). Multiple facets of bacterial porins. FEMS Microbiol. Lett..

[29] Jani A.J., Cotter P.A. (2010). Type VI secretion: Not just for pathogenesis anymore. Cell Host Microbe.

[30] Blondel C.J., Jiménez J.C., Contreras I., Santiviago C.A. (2009). Comparative genomic analysis uncovers 3 novel loci encoding type six secretion systems differentially distributed in *Salmonella* serotypes. BMC Genom..

[31] Weber B., Hasic M., Chen C., Wai S.N., Milton D.L. (2009). Type VI secretion modulates quorum sensing and stress response in *Vibrio anguillarum*. Environ. Microbiol..

[32] Aschtgen M.S., Bernard C.S., De Bentzmann S., Lloubès R., Cascales E. (2008). SciN is an outer membrane lipoprotein required for type VI secretion in enteroaggregative *Escherichia coli*. J. Bacteriol..

[33] Gerth U., Krüger E., Derré I., Msadek T., Hecker M. (1998). Stress induction of the *Bacillus subtilis* clpP gene encoding a homologue of the proteolytic component of the Clp protease and the involvement of ClpP and ClpX in stress tolerance. Mol. Microbiol..

[34] Raju R.M., Jedrychowski M.P., Wei J.R., Pinkham J.T., Park A.S., O’Brien K., Rehren G., Schnappinger D., Gygi S.P., Rubin E.J. (2014). Post-translational regulation via Clp protease is critical for survival of *Mycobacterium tuberculosis*. PLoS Pathog..

[35] Raju R.M., Unnikrishnan M., Rubin D., Krishnamoorthy V., Kandror O., Akopian T.N., Goldberg A.L., Rubin E.J. (2012). *Mycobacterium tuberculosis* ClpP1 and ClpP2 function together in protein degradation and are required for viability in vitro and during infection. PLoS Pathog..

[36] Burtnick M.N., Brett P.J. (2013). *Burkholderia mallei* and *Burkholderia pseudomallei* cluster 1 type VI secretion system gene expression is negatively regulated by iron and zinc. PLoS ONE.

[37] Ahmad A.A., Askora A., Kawasaki T., Fujie M., Yamada T. (2014). The filamentous phage XacF1 causes loss of virulence in Xanthomonas axonopodis pv. citri, the causative agent of citrus canker disease. Front. Microbiol..

[38] Cui Z.Q., Ibrahim M., Yang C.L., Fang Y., Annam H., Li B., Wang Y.L., Xie G.L., Sun G.C. (2014). Susceptibility of opportunistic burkholderia glumae to copper surfaces following wet or dry surface contact. Molecules.

[39] Lou M.M., Zhu B., Muhammad I., Li B., Xie G.L., Wang Y.L., Li H.Y., Sun G.C. (2011). Antibacterial activity and mechanism of action of chitosan solutions against apricot fruit rot pathogen *Burkholderia seminalis*. Carbohydr. Res..

[40] Slutzki M., Barak Y., Reshef D., Schueler-Furman O., Lamed R., Bayer E.A. (2012). Indirect ELISA-based approach for comparative measurement of high-affinity cohesin–dockerin interactions. J. Mol. Recognit..

[41] Yang Q., Yan L., Gu Q., Ma Z. (2012). The mitogen-activated protein kinase kinase kinase BcOs4 is required for vegetative differentiation and pathogenicity in *Botrytis cinerea*. Appl. Environ. Microbiol..

